# Proteasome Modulator 9 SNPs are linked to hypertension in type 2 diabetes families

**DOI:** 10.1186/1475-2840-10-77

**Published:** 2011-08-28

**Authors:** Claudia Gragnoli

**Affiliations:** 1 Laboratory of Molecular Genetics of Complex and Monogenic Disorders, Department of Medicine and Cellular & Molecular Physiology and Biostatistics, Penn State University and M. S. Hershey Medical Center, Hershey, PA, USA; 2 Center for Biotechnology and Department of Biology, Temple University's College of Science & Technology, Philadelphia, PA, USA; 3 Molecular Biology Laboratory, Bios Biotech Multi-Diagnostic Health Center, Rome, Italy

## Abstract

**Background:**

Chromosome 12q24 was recently associated with hypertension. Proteasome Modulator 9 (*PSMD9*) lies in the 12q24 locus and is in linkage with MODY3, type 2 diabetes (T2D), microvascular and macrovascular pathology, carpal tunnel syndrome, and hypercholesterolemia in Italian families.

**Aims:**

Our goal was to determine whether *PSMD9 *is linked to elevated blood pressure/hypertension in T2D families.

**Methods:**

We characterized the Italian T2D families' members for presence and/or absence of elevated blood pressure (≥ 130/80) and/or hypertension. The phenotypes were described as unknown in all cases in which the diagnosis was either unclear or the data were not available for the subject studied. We tested in the 200 Italians families for the presence of the linkage of the *PSMD9 *T2D risk single nucleotide polymorphisms (SNPs) *IVS3+nt460 A > G*, *IVS3+nt437 T > C *and *E197G A > G *with elevated blood pressure/hypertension. The non-parametric linkage analysis was performed for this qualitative phenotype by using the Merlin software; the Lod score and correspondent P-value were calculated. Parametric linkage analysis was also performed. For the significant linkage score, 1000 replicates were run to calculate the empirical P-value.

**Results:**

The *PSMD9 *gene SNPs studied are in linkage with elevated blood pressure/hypertension in our Italian families.

**Conclusions:**

We conclude that the *PSMD9 *gene and/or any variant in linkage disequilibrium with the SNPs studied contribute to the linkage to hypertension within our family dataset. This is the first report of *PSMD9 *linkage to hypertension within the 12q24 locus.

## Introduction

A recent study has shown that changes in retinal vascular caliber are linked to the chromosome 12q24 locus in a large Caucasian population [1]. Microcirculation is important in determination of hypertension [1] and retinal vascular changes reflect early microvascular disease and predict cardiovascular events. In two independent samples, the locus 12q24 was also associated with coronary heart disease and hypertension [1]. Thus, the chromosome 12q24 locus carries at least a gene contributing to hypertension. In this locus lies Proteasome Modulator 9 (*PSMD9*), a coactivator of insulin gene transcription, which is highly expressed in pancreatic islets [2]. PSMD9 is a ubiquitous protein; therefore its biological role may be broad. *PSMD9 *overexpression cause beta-cell dysfunction and contribute to type 2 diabetes (T2D) [2]. We reported that *PSMD9 *may rarely cause T2D by unique mutations [3]. We identified a significant linkage of the *PSMD9 A/T/G *haplotype to late-onset T2D, with the strongest evidence under the recessive model [4], and to MODY3 under the additive model [5] in Italians. The contribution of intronic variants to complex disorders is an accepted concept. We recently reported a linkage of the *PSMD9 *T2D risk single nucleotide polymorphisms (SNPs) with T2D-nephropathy [6], T2D-neuropathy [7], retinopathy [8], carpal tunnel syndrome [9], hypercholesterolemia [10], and macrovascular pathology [11]. Given the reported linkage data of *PSMD9 *within the locus 12q24, and the evidence of linkage with microcirculation within the same locus [1], *PSMD9 *is a candidate gene for hypertension. Further, as *PSMD9 *is linked to T2D, it should be screened to identify any inheritance contributing factors to the T2D-associated phenotypes, of which hypertension is a major player.

In the present study, we aimed at testing the *PSMD9 IVS3+nt460*, *IVS3+nt437*, *E197G *T2D risk SNPs for linkage with elevated blood pressure and/or hypertension in our 200 Italian T2D families.

## Methods

### Ethical Statement

the subjects were all recruited from center Italy following the Helsinki declaration guidelines. Subjects gave written informed consent. The Penn State College of Medicine Ethical Committee approved the study.

### Families

We recruited 200 Italian T2D affected siblings and extended families, including also unaffected members. The families originate from the center of Italy. The members are at least three generations Italians. Identical twins were excluded. These T2D families have been helpful in the whole or as unrelated T2D cases in identifying or excluding diabetes risk genes and/or variants in previous studies [12-24]. We characterized the Italian families for presence/absence of elevated blood pressure/hypertension [presence is considered by blood pressure ≥ 130/80 mmHg in drug-naïve patients or by use of anti-hypertensive medication(s)]. Phenotype is described as unknown if diagnosis is unclear or data are lacking. Most subjects with T2D have elevated blood pressure and few subjects without T2D have elevated blood pressure. The total of the subjects available for the analysis including the ungenotyped parents is 928 (443 founders, 485 non-founders; 467 females, 461 males) with an average family members of 4.62. The diagnostics of hypertension is present in 373 genotyped individuals, with a prevalence of 94.6%.

### Sequencing

Via PCR, we amplified the *IVS3 PSMD9 *region containing the *+nt460 A > G *and *+nt437 C > T *SNPs and the exon 5 coding region containing the *E197G A > G *SNP with specific primers in the affected and unaffected family members. We directly sequenced the PCR products, status post-purification via EXOSAP-IT, on an automated ABI 3730 Sequencer.

### Statistical Analysis

We tested in the 200 Italian families for linkage of the *PSMD9 *SNPs with elevated blood pressure/hypertension. Both non-parametric and parametric linkage analysis for the qualitative phenotype were performed for the three SNPs via Merlin software [25]. Allele frequencies were calculated from the data [25]. Merlin analysed all genetically informative families (n = 129; total subjects = 596) within this dataset, depending on the presence of both genotypes and hypertension phenotype in families whose structure, given the data, was amenable to the linkage tests. The families analysed by Merlin are representative of the population. We previously reported that the *PSMD9 *SNPs *IVS3+nt460*, *IVS3+nt437*, *E197G *are in strong linkage disequilibrium (LD) [3]. The following parameters were used for the parametric linkage analysis based on the SNPs-cluster, thus eliminating the LD inflation of the linkage signal: disease allele frequency 0.25, dominant model with penetrance for homozygous non-risk allele 0.31 (equal to the prevalence of hypertension in the Italian population), for heterozygous risk allele 1.00, for homozygous risk allele 1.00; recessive model with penetrance for homozygous non-risk allele 0.31, for heterozygous risk allele 0.31, for homozygous risk allele 1.00; additive model with penetrance for homozygous non-risk allele 0.31, for heterozygous risk allele 0.45 and for homozygous risk allele 0.90. All results are reported as LOD scores calculated by Merlin.

For each test performed, to exclude the presence of any false positive in our results, we calculated how many times similar P-values were expected by chance in 1,000 replicates of simulations by using the gene dropping method: this analysis replaces real data with simulated data, while maintaining the pedigree structure, allele frequencies and recombination fraction. These datasets are generated under the null hypothesis of no linkage.

## Results

The results of the non-parametric and parametric linkage analysis performed for the qualitative phenotype hypertension are reported in table 1. Both analytical methods have reported significant results with the most significant being for the non-parametric model. The *PSMD9 *SNP *IVS3+nt460 *is slightly more significant than the other two SNPs. Under the parametric model analysis, the most significant is the dominant model. The simulation analyses of 1,000 replicates have excluded false positives and establish the validity of the real data.

**Table 1:** Non-parametric and Parametric Linkage Analysis of Hypertension in the 200 Italian Families by Merlin software.

Phenotype	Prevalence	Families	Lod Score	P	Empirical P
**Non-Parametric**	94.60%	129	3.32	0.00005	0.001

**Dominant Model; SNPs Cluster**	94.60%	129	2.282	0.001202	0.000

**Recessive Model; SNPs Cluster**	94.60%	129	1.979	0.002561	0.000

**Additive Model; SNPs Cluster**	94.60%	129	0.926	0.039048	0.000

Prevalence = phenotype prevalence among the family subjects studied; Families = families number analyzed; Lod score = derived from the non-parametric linkage analysis or the parametric analysis cluster-based by Merlin; P = p-value; Empirical P = p-value derived from 1000 replicates by using the gene dropping method. Hypertension is indicated as presence of blood pressure ≥ 130/80 in absence of medications or presence at least of one anti-hypertensive drug.

## Discussion

Our analysis show that the *PSMD9 IVS3 +nt460 A > G *and *+nt437 C > T *and exon 5 *E197G A > G *SNPs studied and/or any variants in LD with them are in linkage with elevated blood pressure/hypertension in our 200 Italian families. The *PSMD9 *SNPs studied contribute to the linkage of the reported phenotype. It is possible that the recessive and additive models appear less significant than the dominant model, as the disease penetrance value given for the heterozygous state under both models, and for the homozygous state under the additive model, is inferior to 1. Thus, some of the power of the data may be lost. On the other hand, recessive and additive models are still significant as the variants in homozygous state are likely inherited by the affected family subjects more often than expected by the null hypothesis of no linkage and thus contribute to the linkage signal. The limitation of our study is that the linkage may capture the signal from the potential co-inheritance of genetic variants linked to another phenotype and to the underlying T2D that is commonly shared with hypertension; however, as T2D patients have high prevalence of hypertension, virtually all linkage studies performed in T2D may as well mask a linkage with hypertension. In fact, when we statistically analyze only a single phenotype, we cannot protect the results from any potentially known and unknown associated factor with the phenotype under study and from the co-inheritance of genetic variants related to that factor underlying the linkage signal.

The strength of our study resides in the significant data both at the level of non-parametric analysis, which is not vulnerable to miscalculation of allele frequencies and penetrance values, as well as at the level of the parametric analysis, which may suffer from not perfect estimates of allele frequencies and penetrance values.

However, the best statistical power test is the simulation empirical P-value, and in this study all empirical P-values have excluded the possibility of false positives in our data analysis.

These findings have not yet been confirmed in other ethnicities.

## Conclusions

The *PSMD9 *SNPs *IVS3+nt460*, *IVS3+nt437*, *E197G *are significantly linked to elevated blood pressure/hypertension in the Italian T2D family dataset.

## Abbreviations

PSMD9: Proteasome Modulator 9; SNP: single nucleotide polymorphism; T2D: type 2 diabetes; MODY3: maturity-onset diabetes of the young 3; IVS: intervening sequence (intron)

## Competing interests

The authors declare that they have no competing interests.

## Authors' contributions

CG conceived and designed the study, collected the clinical information, performed the statistical analysis and drafted the manuscript. CG also read and approved the final manuscript.
